# Microencapsulated Bilberry and Chokeberry Leaf Extracts with Potential Health Benefits

**DOI:** 10.3390/plants12233979

**Published:** 2023-11-27

**Authors:** Snežana Kuzmanović Nedeljković, Milica Radan, Nada Ćujić Nikolić, Zorana Mutavski, Nemanja Krgović, Smilja Marković, Tatjana Stević, Jelena Živković, Katarina Šavikin

**Affiliations:** 1 Institute for Medicinal Plants Research Dr Josif Pančić, Tadeuša Košćuška 1, 11000 Belgrade, Serbia; 2 Institute of Technical Sciences of SASA, Knez Mihailova 35/IV, 11000 Belgrade, Serbia; smilja.markovic@itn.sanu.ac.rs

**Keywords:** bilberry leaves, chokeberry leaves, microencapsulation, spray drying, hypoglycemic activity, antimicrobial activity, photoprotective activity

## Abstract

The aim of the research was to develop microencapsulated powders of bilberry and chokeberry extracts via the spray drying technique. Two biopolymers, pectin alone and in combination with HP-β-CD, were used to preserve the antioxidant, hypoglycemic, photoprotective, and antimicrobial bioactivity of the berry leaf extracts. Moreover, the formed powders were characterized in terms of technological, chemical, and several biological properties. The obtained micro-sized powders (mean average particle diameter from 3.83 to 5.94 µm) demonstrated a process yield of up to 73%. The added biopolymers improved the flowability and cohesive properties of the powders and increased their thermal stability to 170 °C. The total content of polyphenolics in the powders ranged from 323.35 to 367.76 mg GAE/g DW for bilberry and from 186.85 to 227.59 mg GAE/g DW for chokeberry powders; meanwhile, chlorogenic acid was the predominant compound in powders. All samples showed stronger α-glucosidase inhibitory activity (IC50 values ranged from 5.00 to 19.59 µg/mL) compared with the reference standard. The study confirmed that spray drying is a suitable method for the preservation of the polyphenolic-rich extracts, while the addition of carriers has a positive effect on the improvement of microencapsulated powders’ properties.

## 1. Introduction

Globally, non-communicable diseases are responsible for 74% of all deaths, which is the reason that they are recognized as a major cause of death by the World Health Organization. Among them, diabetes ranks fourth, taking approximately two million lives annually [1]. The westernization of diets, obesity, and metabolic syndrome are spreading globally and are most notable among low-income world regions [2]. Taking this into account, as well as the rise of physical inactivity and environmental and genetic factors that may contribute to the progression of insulin resistance and the development of type 2 diabetes, it is not surprising that by the year 2040, it is estimated that type 2 diabetes will affect about 642 million people on the global level [3]. Both hyperglycemia and oxidative stress are linked with the etiology and pathophysiology of diabetes [3]; therefore, the discovery of novel products with hypoglycemic and antioxidant potential would be a step forward in overcoming this progressive disease. The additional antimicrobial potential could be considered, according to the higher rate of microbial infections and the tendency to develop complications in diabetic patients compared with non-diabetic ones, especially skin changes and diabetic wounds [4]. The most common and complex skin condition associated with hyperglycemia-related diseases is diabetic foot ulcer (DFU), which leads to limb amputation for about 50–70% of diabetic patients [5,6]. Infections, which occasionally have a polymicrobial foundation, are the leading cause of amputation in DFU [5,7]. Antimicrobial resistance and the synergistic effect of these microorganisms represent the core of treatment failure [5]; therefore, there is a growing interest in finding new therapeutic options. Last but not least, elevated glucose levels and oxidative stress induce the excessive production of advanced glycosylation end products (AGEPs), which appear to be the main cause of skin aging during life [8]. Those products are also caused by the effect of UV radiation on the skin [8].

While there is an excessive need for new therapeutic and preventive options, medicinal plants may be a source of plant-derived bioactive components that are potentially valuable to the healthcare system [9]. Phenolic compounds found in medicinal plants were proven to be effective antioxidants that prevent the propagation of free radicals, and their ingestion has been associated with lowering the risk of diabetes [10]. Red fruits from the Rosaceae and Ericaceae families have been well known for beneficial effects mediated by polyphenols, but berry plant leaves, which have not been investigated as frequently, also represent a valuable source of these precious bioactive compounds [10,11,12].

*Aronia melanocarpa* (Michx.) Elliot (chokeberry) and *Vaccinium myrtillus* (bilberry), are the representatives of Rosaceae and Ericaceae families. *Vaccinium* spp. is widespread in Europe, Asia, and North America, and the *Aronia* genus is dominantly native to North America [13,14]. Recently, chokeberry and bilberry leaves entered the focus of scientific research due to their chemical composition. High levels of quercetin derivatives and chlorogenic acid, in addition to the presence of different phenolic components, have been reported in the leaves of these two species [15,16,17,18,19,20]. Accordingly, a few studies pointed out that chokeberry leaves possess different biological activities, such as antioxidant, anti-neurodegenerative, hypoglycemic, and anticancer activity [21,22,23,24]. Likewise, antibacterial, anti-inflammatory, antioxidant, and blood glucose- and lipid-lowering effects have been detected in *Vaccinium* genus leaves [18,25,26,27].

The extraction of bioactive components can be performed by different techniques. Percolation, one of the conventional methods, has been widely used for the preparation of liquid extracts [28,29]. However, dried dosage forms of herbal products are preferred over liquid ones due to better stability and more comfortable use [30]. To preserve valuable bioactive compounds and produce stable dried extracts with good technological characteristics, different techniques have been applied, and one of them is spray drying [31,32]. It is a widely used, well-established process that enables powdered microcapsules to be obtained from liquid extracts. The selection of the proper carrier material is crucial since it affects yield, technological characteristics, chemical composition, and biological activity [32]. Carbohydrates, lipids, and proteins or their derivatives are commonly used carriers for the encapsulation of bioactive compounds [33]. Pectin, a hydrophilic anionic polysaccharide, is usually obtained from algae cell walls or the skin and pulp of citrus fruits and apples. Pectin can be cross-linked with bioactive molecules to ensure its role as a carrier in the microencapsulation process [34], and it is particularly interesting because of its proven hypoglycemic, antioxidant, antibacterial, anti-inflammatory, immunoregulatory, and antitumor potential [35]. Also, cyclodextrins are relatively newly applied encapsulating agents. These molecules have a specific structure with a central hydrophobic cavity and a hydrophilic external part that enables the formation of inclusion complexes with a significant number of organic and inorganic molecules [36]. Within this large group of oligosaccharides, β-cyclodextrin stands out as the most accessible and affordable. Additionally, its hydroxylated product, 2-hydroxypropyl-β-cyclodextrin (HP-β-CD), takes primacy with its improved solubility in water compared with the non-hydroxylated form [37]. The combinations of conventional and novel carriers could provide a good strategy for producing stable dried extracts [38].

While the microencapsulation of anthocyanins and polyphenols from juice, wine, and liquid fruit extracts of the mentioned plants has been implemented in various studies to preserve bioactive compounds or make their application easier [39,40,41,42,43,44,45], information on the preservation of polyphenols from the leaves is still scarce [46]. To our knowledge, this is the first study to investigate the effects on the preservation of *A. melanocarpa* and *V. myrtillus* polyphenols originating from the leaves using pectin and HP-β-CD as carriers.

Considering the current concern about diabetes, the aim of our study was to develop a feasible process for the preparation of stable, dried extracts of chokeberry and bilberry leaves using pectin as an encapsulating carrier alone and in combination with HP-β-CD with preserved beneficial hypoglycemic, antioxidant, antimicrobial, and photoprotective effects. The obtained extracts were evaluated in terms of their chemical, biological, and technological properties, as well as their stability.

## 2. Results and Discussion

### 2.1. Analysis of Technological Characteristics of Microencapsulates

#### 2.1.1. Powder Yield

The liquid extract (feed) was obtained using 50% ethanol as a solvent. The powder yield (PY) demonstrates the economic profitability and successful achievement of the drying process, and a PY of over 50% is associated with an efficient drying technique [47,48]. In our study, the value of the PY varied between 61.99 and 67.86% for bilberry powders and from 67.87 to 73.27% for chokeberry powders. For both encapsulated extracts, the addition of pectin increased the powder yield, while the addition of HP-β-CD had a positive effect on the yield of the bilberry powders. HP-β-CD’s effect of increasing the yield from 38.4% to 83.8% for the encapsulation of grape cane extracts for was also noted by Escobar-Avello et al. (2021) [49]. For the encapsulated bilberry leaf extracts, the highest yield of 67.86% was obtained with the addition of a carrier blend (BPCD), while for the chokeberry leaf extracts, the yield of 73.27% was quantified with sample CP (Table 1). In favor of using the pectin/HP-β-CD combination, Sansone et al. (2011) concluded that the combination of pectin with other conventional and non-conventional carriers improved the PY of *Fadogia ancylantha* (Makoni tea), *Melissa officinalis* (lemon balm), and *Tussilago farfara* (coltsfoot) encapsulates [50].

#### 2.1.2. Moisture Content

In all obtained powders of bilberry and chokeberry leaf extracts, the moisture content was less than 5%; it varied between 3.35 and 3.91% for the bilberry powders and between 2.95 and 3.41% for the chokeberry powders, which corresponds to their quality and quantity of bioactive ingredients [51]. In general, the moisture content is one of the most important physical characteristics of the powders, due to the microbiological stability of the future products. Namely, increased moisture in the environment favors the development of microorganisms and promotes the deterioration of powders [52]. It can be noted (Table 1) that the addition of HP-*β*-CD contributed to a lower moisture content in the obtained powdered extracts, which is in accordance with our previous results [38].

#### 2.1.3. Bulk and Tapped Densities, Carr Index, and Hausner Ratio

Bulk density, a quality parameter of dried extracts, is important for final product formulations as well as storage, packaging, and distribution conditions [49,53]. In our samples, the values ranged from 0.21 to 0.29 g/mL for bilberry leaf extracts and from 0.20 to 0.27 g/mL for chokeberry leaf extracts. The bulk density of powders produced with carriers had significantly higher values compared with powders without the addition of carriers. Carrier type had an impact on bulk density, which was reported in a study conducted by Martinić et al. (2022) on dandelion leaf extracts [54]. Powders with a higher moisture content have a higher bulk density according to Caliskan et al. (2016) [55].

Generally, for the tapped densities no significant differences were noted between examined leaf powders (Table 1), while only B had a slightly lower value (0.31 g/mL).

Biopolymer inclusion improved the flowability and cohesiveness properties of the powders; meanwhile, pectin was a more efficacious carrier (Table 1).

#### 2.1.4. Rehydration

Rehydration is a significant powder characteristic because the most common application is dissolving the powder in water before oral administration [53,56]. BPCD and CPCD showed the shortest rehydration times (29.27 and 21.48 s, respectively). The addition of HP-β-CD significantly reduced rehydration time, which is in line with the data from the literature [36,40]. Conversely, pectin brought down the solubility time of the obtained powders, which is in accordance with pectin’s prolonged release [57]. The results can be found in Table 1.

### 2.2. Physical Characterization of Microencapsulates

#### 2.2.1. Particle Size Distribution

**Figure 1:**
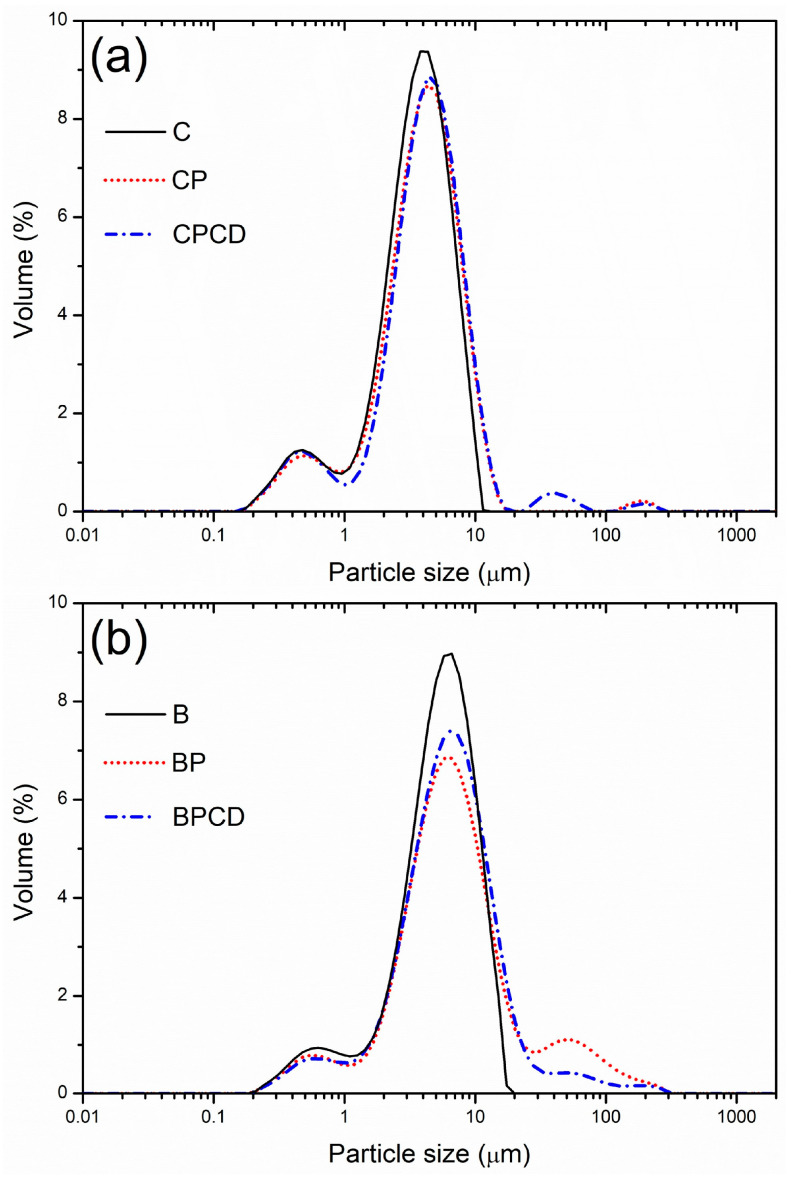
Particle size distribution of bilberry and chokeberry leaf extracts and their microencapsulates: (**a**) C—chokeberry extract; CP—chokeberry extract with pectin; CPCD—chokeberry extract with pectin and HP-β-CD; (**b**) B—bilberry extract; BP—bilberry extract with pectin; BPCD—bilberry extract with pectin and HP-β-CD.

Since particles produced by the spray-drying technique have the potential to come in different size ranges, the size distribution of chokeberry and bilberry extracts loaded in biopolymers was established and is presented in Table 2. The modal distribution of the obtained spray-dried powders was determined (Figure 1) and showed three dominant distinct peaks. The particle size diameters of the spray-dried chokeberry extract ranged widely from 0.99 (d_10_) to 7.41 µm (d_90_), with a mean average diameter d_50_ of 3.83 µm; the lowest particle diameters were exhibited among chokeberry microencapsulates, which were highly uniform. The microencapsulates produced with pectin and HP-β-CD promoted a non-significantly higher mean diameter. Generally, carrier addition resulted in increased particle size compared with that of pure dried chokeberry extract, which is in accordance with the previously reported results of bilberry juice microencapsulation by Wilkowska et al. (2016) and green tea extract by Pasrija et al. (2015) [36,58]. The PDIs of the produced powders were characterized by low span values, indicating good particle uniformity; the most uniform and homogenous extract was the spray-dried chokeberry extract, followed by CP and CPCD.

Spray-dried bilberry extract and its microencapsulates can be evidenced by slightly higher particle size diameters. The diameters of the microparticles varied from 1.34 (d_10_) for B to 31.97 µm (d_90_) for BP, with the mean average diameter d_50_ from 4.74 to 5.94 µm for all bilberry extract-loaded microencapsulates (Table 2). The highest particle diameters and the highest span value were obtained with pectin as a carrier. The PDI differences among bilberry microencapsulates may be attributed to dissimilar polymer properties [59]. The observed trend of increased particle size and decreased particle uniformity is linked to the tendency of pectin to swell and produce higher-diameter particles, which is in accordance with our previous study on *Gentiana asclepiadea* L. microencapsulation [60]. HP-β-CD addition to the pectin–extract complex decreased the particle size and increased the particle homogeneity determined by PDI values. According to the literature, Lim and Dolan (2011) obtained bilberry extract in a maltodextrin carrier with a higher particle size than that of the bilberry microparticles produced in our study [61].

Both pure chokeberry and bilberry extracts exhibited good particle uniformity with low mean particle diameter, confirming that spray-drying performances were appropriate for this kind of herbal extract. In accordance with Gawalek et al. (2017), the mean particle size of spray-dried chokeberry juice at even higher inlet temperatures ranged from 20 to a maximum of 40 µm [62], and in our previous study on microencapsulated chokeberry juice and extract, the particle size was in the range of 4.27 to 11.07 µm [31]. Righi da Rosa et al. (2018) reported spray-dried bilberry extract microparticles from 12.8 to 20 µm [63]. The mean particle size of the spray-dried chokeberry and bilberry micro-powders reached a maximum of 5.94 µm, indicating the formation of small and uniform particles.

#### 2.2.2. FTIR Spectroscopy

**Figure 2:**
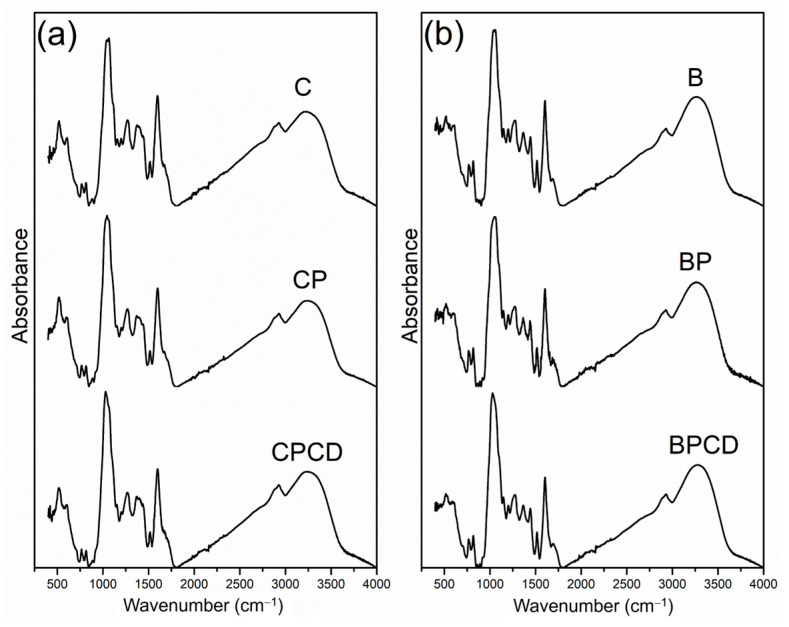
FTIR spectra of bilberry and chokeberry leaf extracts and their microencapsulates: (**a**) C—chokeberry extract; CP—chokeberry extract with pectin; CPCD—chokeberry extract with pectin and HP-β-CD; (**b**) B—bilberry extract; BP—bilberry extract with pectin; BPCD—bilberry extract with pectin and HP-β-CD.

FTIR analysis is a widely used technique for microencapsulated system characterization and can rapidly and simply determine the composition and changes during the microencapsulation process. The FTIR spectra of the microencapsulated chokeberry and bilberry extracts and the utilized biopolymers are presented in Figure 2. The spectra of the microencapsulated chokeberry leaf extract contained a band around 500 cm^−1^ (although the fingerprint region between 500 and 1000 cm^−1^ is generally not useful), which tends to correspond to sugar cycles [64]. Absorption bands in the region of 1000 cm^−1^ in both extracts and their microencapsulates were assigned to C-H and C-O stretching vibrations of the aromatic rings, which result from the aromatic compounds of phenolics as well as the carbohydrate nature of biopolymers [31,38,65]. The spectral peak region from 1500 to 1600 cm^−1^ is associated with C=C aromatic stretching vibrations from extracts polyphenols, notable in both spray-dried extracts and their microencapsulates; meanwhile, COOC carboxylate vibrations originated from pectin molecules [66].

The most characteristic spectra of both microencapsulated extracts, represented by the highest absorption band between 3600 and 3000 cm^−1^, originated from O–H (hydroxyl) groups that originated from many polyphenolic compounds, corresponding to the literature [31,67,68]. These absorption peaks are equally evident across all examined spectra, regardless of the dilution effect of the carriers used for the microencapsulation process. The small absorbance peak at 2900 cm^−1^ has been assigned to C-H stretching [49,69].

The FTIR analysis likewise represents a method that allows the identification of the possible interactions between the polymers and phytochemicals. In our study, all examined samples were characterized by similar spectra, with no visual characteristic differences between the extract-loaded microparticles. This property specifies that the chemical bonds characteristic of the functional ingredients of chokeberry and bilberry extracts and the biopolymers used are preserved and that the microencapsulation could be attributed to physical interactions rather than chemical ones [70]. Therefore, the spray-drying performance did not cause structural changes in the polymer matrix and the extracts. Insignificant alterations in intensity were noted among the samples, implying a microencapsulation of phenolic compounds in two different biopolymers without interactions among them.

#### 2.2.3. Differential Scanning Calorimetry (DSC)

**Figure 3:**
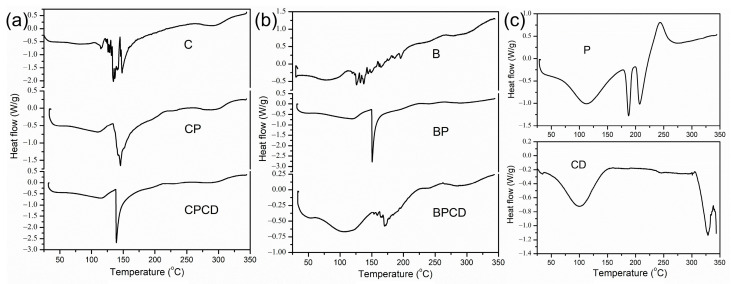
DSC thermograms of bilberry and chokeberry leaf extracts: (**a**) C—chokeberry extract; CP—chokeberry extract with pectin; CPCD—chokeberry extract with pectin and HP-β-CD; (**b**) B—bilberry extract; BP—bilberry extract with pectin; BPCD—bilberry extract with pectin and HP-β-CD; (**c**) P refers to pectin and CD to HP-β-CD.

The thermal stability was performed by DSC analysis for the spray-dried extracts, biopolymers, and extract–biopolymer matrices. The degradation temperatures of the observed powders could be affected by several different factors, such as chemical composition, the molecular weight of the carriers and phytochemicals in the extract materials, and moisture content [71]. The characteristic thermal transformations of chokeberry and bilberry extracts are shown by the DSC thermograms in Figure 3, while the peak transition temperatures and enthalpy changes (∆H) are presented in Table 3.

From the initial temperature (20 °C) to about 55 to 60 °C, there was no observed thermal activity of the chokeberry powders. The induced thermal degradation in the pure chokeberry powder was shown by three temperature changes, with a peak maximum reached at about 135 °C, while the final degradation was observed around 145 °C (Figure 3a). The enthalpy changes of this sample reached the highest value around 102 J/g. Carrier addition in chokeberry extract reduced unclearly defined temperature changes to two clearly defined ones. Additionally, in all three chokeberry microencapsulates, at a temperature of around 150 °C, strong endothermic peaks were detected; this was a consequence of the carbohydrates’ thermal transition, which was more pronounced with the addition of carriers, especially a combination of carriers. Since the added biopolymers predominantly had carbohydrate fractions, it can be claimed that the signals around 150 °C on the DSC curves originated from carbohydrate fiber decomposition [72].

For bilberry extract, heat transformation started at 45 °C, underwent two small phase transitions, and finished at around 142 °C. Added carriers prevented early temperature degradation and moved the start of temperature degradation for 20 °C. Temperature changes with carrier addition reduced and clearly defined the temperature changes in comparison with pure bilberry extract. It is worth mentioning that the peak degradation temperature reached the highest value for bilberry extract embedded in carrier blends at 170 °C, in comparison with pure dried extract at 137 °C, improving BPCD thermal stability, probably due to the complex formation of encapsulated extract and CD in the carrier cavity [73]. In fact, by analyzing the thermal stability of spray-dried powders, we showed that carriers could protect the spray-dried particles against temperature degradation [38], probably by promoting the formation of a thin film around the droplets, making them more stable [41,72,74]. The DSC analysis of our samples signified that all microencapsulates possessed good thermal stability in the temperature region important for food and pharmaceutical processing, storage, and consumption.

### 2.3. Chemical Analysis of Microencapsulates

#### 2.3.1. Analysis of Total Phenolics

The content of total polyphenolic compounds in the bilberry and chokeberry leaves microencapsulates were in the range of 323.35 to 367.76 mg GAE/g DW and 186.85 to 227.59 mg GAE/g DW, respectively (Table 4). The highest yield of total polyphenols was measured in the carrier-free bilberry and chokeberry leaf extracts (B and C). However, slightly lower values were observed in the extracts with pectin (BP and CP) and pectin/HP-β-CD (BPCD and CPCD). The results are supported by the fact that encapsulation with carriers is accompanied by the reduced content of leaf extract due to the addition of 10% pectin or 10% pectin and 5% HP-β-CD in comparison with the samples that possessed pure extract without carriers. Previous investigations reported in the literature have shown that the total phenolic content (TPC) of bilberry leaf extracts ranges from 95.17 to 275.68 mg GAE/g DW, as shown in a study by Xiaoyong et al. (2014) [75], while Wu et al. (2019) identified three cultivars with TPC values over 200.0 mg GAE/g DW [76]. The slightly higher phenolic content in bilberry leaf extracts observed in our study might have been caused by different experimental factors, such as the solvent type and concentration, extraction methods, and plant varieties. The results obtained for chokeberry leaves are in agreement with a previous report on *Aronia melanocarpa*, which ranged between 88.30 and 221.50 mg GAE/g DW [21].

#### 2.3.2. HPLC Analysis of Individual Compounds

Although there are numerous reports on the polyphenolic profile of chokeberry and bilberry fruits, reports on the quantification of phenolic compounds from the leaves of these species are scarce. In this study, the content of chlorogenic acid and quercetin derivatives was analyzed using the HPLC technique, and the results are presented in Table 5. The dominance of these compounds in the leaves of both species has also been shown by other authors [21,77,78,79]. Except in the case of quercetin, the content of the investigated compounds was higher in extracts obtained from bilberry leaves compared with that obtained from chokeberry leaves. At the same time, a slightly higher content of individual polyphenolics was measured in carrier-free extracts. As was the case for total phenolic compounds, the reason for this was the reduced content of the leaf extract due to the addition of carriers (10% pectin or 10% pectin and 5% HP-β-CD). In all the investigated samples, chlorogenic acid was the dominant compound ranging from 48.27 to 54.01 mg/g DW and 34.66 to 40.05 mg/DW in bilberry and chokeberry leaf microencapsulates, respectively. Regarding quercetin derivatives, isoquercetin was dominant, with the content ranging from 37.20 to 40.25 µg/g DW in bilberry extracts and 10.70 to 11.37 µg/g DW in chokeberry. A slightly higher content of quercetin was obtained using pectin alone as a carrier, while in the case of other individual compounds, both carriers achieved comparable results.

### 2.4. Biological Evaluation of Microencapsulates

#### 2.4.1. Radical Scavenging Activity—DPPH Assay

Bilberry and chokeberry bioactive phytochemicals have well-recognized health benefits, including powerful antioxidant and anti-inflammatory properties, particularly those involved in metabolic conditions [80,81]. Herein, the antioxidant activity of the studied leaf extracts was evaluated by DPPH assay, and the obtained results are presented in Table 4. Both bilberry and chokeberry leaf extracts exhibited comparable antioxidant activity to vitamin C (IC_50_: 4.45 ± 0.05 µg/mL), which was used as the standard. The most potent radical scavenging activity was observed for bilberry leaf extract without carrier (9.14 µg/mL), while BP and BPCD exhibited slightly lower potency (11.02 and 10.79 µg/mL, respectively), which could be ascribed to the dilution of the extract. The studied bilberry leaf extracts showed more than twice the antioxidant potential as chokeberry leaf extracts, which exhibited the same trend between samples with and without carriers (Table 4). This finding is consistent with the chemical analysis, showing that chokeberry microencapsulates possess a lower content of total and individual phenolic compounds, especially isoquercetin. Previous studies in this area have suggested that the high antioxidant properties of the berry leaves can largely be attributed to the presence of flavonols and phenolic acids, although this depends not only on the species but also on the harvesting time of the raw material [79,82,83]. In general, the DPPH-scavenging mechanism mainly involves intermolecular hydrogen atom abstractions, leading to the formation of stable DPPH-H molecules [84]. Chlorogenic acid, rutin, hyperoside, isoquercetin, and quercitrin, which predominate in the extracts of bilberry and chokeberry leaves, have been previously reported to possess strong antioxidant capacity, as demonstrated in many in vitro and in vivo tests [85,86,87,88]. Furthermore, several studies have emphasized the higher antioxidant potential of the leaves compared with the fruits of the respective berry species [79,82]. Therefore, it could be concluded that the polyphenol profile of the secondary metabolites in the bilberry and chokeberry leaves determined their antioxidant activity, which was also confirmed by the Pearson correlation analysis. The obtained results demonstrated a significant and very high correlation between the antioxidant activity and the content of total and individual phenolic compounds, as presented in Table 6. Several studies on the bilberry and chokeberry leaf extracts have also shown that these extracts are a significant source of natural antioxidants that could be used for nutraceutical and therapeutic purposes [21,79,89,90]. Bljajić et al. (2017) demonstrated a slightly lower antioxidant capacity of the hydroethanolic extract of *Vaccinium myrtillus* leaves (17.80 µg/mL) compared with the results observed in this study [91]. The strong scavenging activity in the DPPH assay of *Aronia melanocarpa* leaf extract was also confirmed by Cvetanović et al. (2018) (30.45 µg/mL), although the obtained results indicated a lower antioxidant capacity of the extract compared with those presented herein [92].

#### 2.4.2. Evaluation of Hypoglycemic Activity

It is recognized that phytotherapy, besides pharmacological treatment, plays an influential role in the management of type 2 diabetes, a metabolic disorder associated with imbalanced glycemic levels within the reference range [13,93]. To regulate the bioavailability of glucose and suppress hyperglycemia, the inhibition of *α*-amylase and *α*-glucosidase, gastrointestinal enzymes involved in carbohydrate digestion, is a valuable therapeutic approach [93]. In this study, the hypoglycemic potential of the group of powders prepared with bilberry and chokeberry leaf extracts and different carriers by the spray-drying technique was investigated by testing their in vitro α-amylase and α-glucosidase inhibitory activities. The obtained results (Table 7) showed that all bilberry leaf extract and chokeberry leaf extract-based powders exhibited stronger *α*-glucosidase inhibitory activity (IC_50_: 5.00–19.59 µg/mL) than the commercial drug acarbose (IC_50_: 156.64 µg/mL), and a mild inhibition regarding *α*-amylase activity. In both assays, the highest activity was determined in extracts without carries, followed by extracts with pectin and extracts with pectin and HP-β-CD; this can be attributed to an increase in the total carrier amount. However, this slight difference was statistically notable only in the case of *α*-glucosidase inhibitory activity between the samples with or without carriers. Analogously, a study conducted by Vidović et al. (2014) showed that *Satureja montana* dried powder extract prepared by the addition of 50% maltodextrin exhibited lower angiotensin I-converting enzyme activity compared with powders with 10% and 30% of the same carrier [48]. However, the addition of drying carriers is crucial to ensure the stability of the sensitive active components of the herbal preparation, which are responsible for pharmacological properties, during the storage period [60]. In previous studies, a noteworthy *α*-glucosidase inhibitory activity of *V. myrtillus* leaf hydroethanolic extract (IC_50_ 0.29 mg/mL) [91] and *A. melanocarpa* leaf 70% ethanolic extract (IC_50_ 5.49 μg/mL) [21] has been demonstrated, which is in line with our results. Finally, considering chemical composition, caffeoylquinic acid and quercetin derivatives could be considered one of the main α-amylase and α-glucosidase inhibitors present in bilberry and chokeberry leaf extracts [13,94].

#### 2.4.3. Antimicrobial Activity

Microbial resistance to antibiotics has been listed among the most significant problems in human health [95]. With the growing rate of multidrug-resistant bacteria, the effectiveness of therapy is decreasing, and therefore, the search for alternative antimicrobial products has become the priority [96]. Some naturally derived products can be a good source of antimicrobials, given their increasing production in plants as a response to microbial infection [97]. Numerous studies have proven the high antimicrobial potential of bilberry [98,99,100] and chokeberry [101,102], but while the fruits are dominantly investigated, there are fewer results for the leaf extracts.

In this study, the dried leaf extracts of bilberry and chokeberry and their microencapsulates were evaluated for antibacterial and antifungal capacity on the most common microorganisms that can cause foodborne or skin infections. These results are presented as minimum inhibitory concentrations (MICs) in Table 8 and Table 9, while the minimum bactericidal and fungicidal concentrations are presented in Supplementary Table S1.

Among foodborne bacteria, the most resistant Gram-negative bacteria were *S.* Typhimurium and *E. coli*, and the most abundant Gram-positive bacteria were *L. monocytogenes*. However, both bilberry and chokeberry extracts exhibited significant inhibitory activities toward *E. faecalis* (MIC values of 2.5 and 10–15 mg/mL, respectively), in addition to bilberry extract’s activity against *S. flexneri* (MIC = 5–7.5 mg/mL).

In the case of microorganisms that cause skin infections, bilberry and chokeberry plant extracts manifested great antimicrobial potential against Gram-positive bacteria, with MICs of 1.75–2.5 mg/mL and 5–7.5 mg/mL for *S. aureus* and *S. epidermidis*. Good inhibitory activity was detected for *E. coli*, and intermediate activity was observed for *A. brasiliensis.* In this group, *P. aeruginosa* proved to be the most resistant of all, which is not a surprise knowing the many defense mechanisms these bacteria use in their fight with antimicrobials [103].

The antimicrobial activity of bilberry and chokeberry extracts has been investigated by several authors, but the comparison is not absolute due to the different methodologies used to investigate the microbiological potential or prepare extracts, including the final type of leaf extract (dried or liquid consistency). Also, the content of dominant bioactive compounds have a large impact on biological activities. Whereas Tian et al. (2018) detected moderate activity of bilberry extracts on *S. enterica* sv. Tyhpimurium and no activity on *S. aureus* and *E. coli* [89], Stefanescu et al. (2020) detected a significant inhibitory effect on *S. aureus* isolates [102]. Our results are also in accordance with Sadowska et al. (2014), who showed that the MIC for the *S. aureus* ATCC 29213 isolate was 1.5 mg/mL [101]. For chokeberry leaf extract, there are different findings as well. Our results are mainly similar to a study that characterized *S. aureus* as the most sensitive bacteria, while *L. monocytogenes* proved to be the most resistant one [100]. Possibly because of different cell wall structures, fungi were less sensitive to these plant extracts. Stefanescu et al. (2020) reported an MIC of 250 mg/mL for bilberry leaf extract on *C. albicans* [102], while Nohynek et al. (2006) concluded that bilberry fruit extract had no inhibitory effect on this fungus [104]. Liepina et al. (2013) also described chokeberry fruit extracts as good antibacterial agents with no antifungal activity [105].

This study outlines the activity of bilberry and chokeberry dried extracts against multidrug-resistant microorganisms. Both plant extracts contain various polyphenols with antimicrobial potential, so the final activity originates from their synergistic effect [13]. The disintegration of the liposaccharide layers of the outer membrane and cytoplasmic phospholipid bilayer followed by the leakage of macromolecules, along with the anti-adhesion effect and complexation of metal ions, provides the multi-target approach for polyphenols [13,101]. Even small chemical structure variations between the contents of extracts may complicate resistance development, which is essential in the treatment of multi-resistant bacterial strains [106]. Compared with Gram-positive bacteria, Gram-negative strains are usually more resistant to various antimicrobials, including naturally derived products, which may be associated with their more complex cell wall structure that can prevent or slow antimicrobials’ passage through membranes [13].

In this study, the activity of bilberry and chokeberry dried extracts was similar for more resistant strains; however, this difference has been detected for sensitive isolates. Among them, the minimum inhibitory concentrations for Gram-positive bacteria were lower than ones for Gram-negative bacteria. The lower sensitivity of Gram-negative strains can be explained by their more complex structure, which prevents polyphenols from achieving their full activity. Bilberry extracts were generally more enriched with the total phenolic content and had the most polyphenols, which was most noticeable in the case of isoquercetin. That can be associated with their better antimicrobial potential compared with chokeberry extracts. The correlation between antimicrobial activity and polyphenol content was evaluated with bivariate Pearson’s correlation, and the results are presented in Table 10. A statistically significant and high correlation (Pearson’s R > 0.7, *p* ˂ 0.05) was detected for sensitive Gram-positive and Gram-negative bacteria isolates, while for the resistant microorganisms, the significance was not notable.

The purpose of this study was to improve the stability of plant leaf dried extracts and their components by conjugating them with pectin and HP-β-CD. There are different findings about the effects of different carriers on antimicrobial activity. Zhang et al. (2018) described increasing antimicrobial activity of Star anise essential oil encapsulated with HP-β-CD on *R. stolonifer*, *S. cerevisiae*, and *E. coli* [107], and Mundlia et al. (2019) found an improvement in naringenin’s inhibitory activity against *S. aureus*, *S. epidermidis*, and *P. aeruginosa* when it was encapsulated with pectin [108]. However, in a study by Chang et al. (2012), it was concluded that conjugation of ε-polylysine with high-methoxyl pectin led to a decrease in antimicrobial efficacy, which was more noticeable with an increase in the carrier fraction [109]. For bilberry and chokeberry extracts, comparable activity was measured for pure extracts and extracts with pectin alone or with HP-β-CD. Small variations can be explained by the dilution effect. Considering that pure and encapsulated extracts have the same compositions and similar content of total phenols and individual components, comparable antimicrobial activity is a valid outcome. Therefore, it can be concluded that for bilberry and chokeberry leaf extracts, the inhibitory activity against various microorganisms was preserved with encapsulation.

#### 2.4.4. Photoprotective Activity

There are reports that exposure to ultraviolet (UV) radiation, particularly the UVB region (280–320 nm), may lead to different skin-related disorders, such as sunburns and accelerated skin aging [110]. Secondary metabolites of plants, including polyphenols–flavonoids and phenylpropanoids, have been identified as promising naturally occurring photoprotective agents [111]. Therefore, this study evaluated the in vitro photoprotective capacity of bilberry and chokeberry leaf extract-based powders, which are rich in flavonols and the phenylpropanoid derivative (chlorogenic acid), by calculating the sun protection factor (SPF). Generally, a concentration-dependent increase in SPF was observed. The SPF values of the investigated powders were in the range of 1.91–9.74, and they are presented in Table 11. The powders without carriers showed higher SPF values compared with powders with carriers but with no significant difference except when comparing B and BPCD. All tested samples at a concentration of 0.1 mg/mL provided an SPF ≥ 6, which is the minimum degree of UVB protection for sunscreen products [112]. The data on the photoprotective activity of bilberry and chokeberry leaf are scarce. However, matching results were announced by Priyanka et al. (2018), who reported that the SPF values of water and methanol leaf extracts of *Eucalyptus* sp. at a concentration of 100 µg/mL were 12.3 and 12.6, respectively [110]. Moreover, Her et al. (2020) found that, after topical application, water *A. melanocarpa* fruit extract attenuated UVB-induced skin damage (i.e., a thickened epidermis and disrupted collagen) as a result of the protective effects of the main extract compounds—chlorogenic acid and rutin [113]. Overall, the photoprotective activity of bilberry and chokeberry leaf dried powder extracts was in accordance with the total phenolic content, as well as with the content of chlorogenic acid and the individual flavonoid contents.

## 3. Materials and Methods

### 3.1. Plant Material

Chokeberry leaves (*Aronia melanocarpa*, (Michx.) Elliot) were collected from cultivated plants from the experimental field village Grabovac, Serbia, at the beginning of September 2022. The leaves were dried at room temperature and kept in a dark and dry place before further analysis. Bilberry leaves (*Vaccinium myrtillus*, L.) were obtained from the manufacturing sector of the Institute for Medicinal Plant Research “Dr. Josif Pančić”, Belgrade, Serbia. The leaves were kept in a dark and dry place before further analysis.

### 3.2. Chemicals

The following standards and reagents were used: punicalin and punicalagin (Sigma Aldrich, St Louis, MO, USA); gallic and ellagic acid (Extrasynthese, Cedex, France); hydroxypropyl-β-cyclodextrin (Acros Organics, Geel, Belgium); pectin (CPKelco, Großenbrode, Germany),; HPLC-grade formic acid, orthophosphoric acid (Sigma Aldrich, St Louis, MO, USA), and acetonitrile (Merck, Darmstadt, Hesse, Germany); methanol, Folin–Ciocalteu phenol reagent, sodium carbonate, and α-amylase and α-glucosidase enzymes (Sigma Aldrich, St Louis, MO, USA); potato starch solution (Thermo Scientific, Waltham, MA, USA); 3,5-dinitrosalicylic acid (DNS), acarbose, and p-nitrophenyl-α-D-glucopyranoside (Acros Organics, Geel, Belgium); phosphate buffer components: sodium-chloride and sodium-dihydrogen phosphate, anhydrous (Centrohem, Stara Pazova, Srbija); 2,2-diphenyl-1-picrylhydrazyl (DPPH) (Sigma Aldrich, St Louis, MO, USA), and vitamin C (Acros Organics, Geel, Belgium). Ethanol (96%) and distilled water were obtained from the production site of the Institute for Medicinal Plants Research “Dr. Josif Pančić”. Ultrapure distilled water was obtained through the Milli-Q water purification system (Millipore, Molsheim, France).

### 3.3. Preparation of Extracts

The double-percolation technique, using 50% ethanol–water as a solvent, was used for the preparation of the extracts. After the screening analysis, it was determined that 50% ethanol was the optimal solvent for the extraction of polyphenols from chokeberry and bilberry leaves. Percolation was carried out at room temperature at a ratio of drug-to-solvent of 1:5. The obtained extracts were evaporated with a vacuum evaporator until the residual ethanol concentration was below 5%, which was very important due to the subsequent extract drying process. The prepared extracts were stored in a dark bottle in the fridge until the next process.

### 3.4. Microencapsulation by Spray-Drying Method

The obtained liquid extracts of chokeberry and bilberry leaves with an ethanol percentage of less than 5% were spray-dried with and without the addition of biopolymers (carriers). Microencapsulates were obtained using pectin (10%) and a combination of pectin and HP-β-CD (10 and 5%, respectively). The concentrations of the carriers used in experiments were calculated on the basis of the extract’s dry weight. Biopolymers were separately dissolved in extracts before the microencapsulation process; meanwhile, HP-β-CD was dissolved 24 h before the process, enabling the micellization process. The prepared solutions were heated to 40 °C and mixed with a magnetic stirrer with a constant extract homogenization. The feed mixtures were microencapsulated in a LabtexESDTi spray dryer (Labtex, Huddersfield, UK), under the following conditions: inlet temperature, 130 ± 5 °C; outlet temperature, 70 ± 5 °C; liquid feed rate, 11 mL/min; spraying air flow rate, 75 m^3^/h; and atomization pressure, 2.5 bar. Before the analysis, the powders were stored in glass bottles in desiccators at room temperature.

### 3.5. Analysis of Technological Characteristics of Microencapsulates

#### 3.5.1. Powder Yield

The powder yield (PY) was calculated according to Equation (1) as the ratio between the obtained mass of the dried powder (m_p_) from the collection vessel of the device and the mathematically calculated expected mass of the powder (the mass of the dry residue of the collected extract + carrier mass, m_ep_):

PY (%) = m_p_/m_ep_ × 100, (1)

#### 3.5.2. Moisture Content

The moisture content (MC) of each obtained powder was analyzed gravimetrically by drying at 105 °C to a constant mass using a halogen moisture analyzer HB43-s (Mettler Toledo). The moisture content was calculated according to Equation (2), where m_m_ is the mass of moisture in the analyzed powder and m_s_ is the mass of the sample before drying:

MC (%) = m_m_/m_s_ × 100, (2)

#### 3.5.3. Bulk and Tapped Densities, Carr Index, and Hausner Ratio

The determination of bulk density (ρ_b_) was performed according to the method previously described by Vidović et al. (2014) with minor modifications [48]. A sample mass of 1 g was placed in a graduated glass cylinder of a certain volume (5 mL) and shaken for 5 min with stirring at 300 rpm. After 5 min of mixing, the volumes of dried powder in the glass cylinder were measured directly from the cylinder. The bulk density was calculated as the ratio of the powder mass and the measured volume of dried powder and expressed as grams of the dried powder’s mass per milliliter (g/mL).

The tapped density (g/mL) was directly measured by reading the volume from the cylinder after tapping the powders 120 times.

The flowability and cohesiveness values of the powders were determined in terms of the Carr index (CI) and Hausner ratio (HR), respectively, calculated using Equations (3) and (4):

CI = (ρ_t_ − ρ_b_)/ρ_t_ × 100, (3)

HR = ρ_t_/ρ_b_, (4)

#### 3.5.4. Rehydration and pH

The rehydration time of powders can be considered the period necessary for complete dissolution in water at room temperature. It was measured as the time taken for the full reconstitution of 1 g of powder in 50 mL of water on a magnetic stirrer, expressed in seconds (s). In each dissolved powder, pH values were determined using a pH meter (Hanna HI 99161, Portugal).

### 3.6. Physical Characterization of Microencapsulates

#### 3.6.1. Particle Size Distribution

The particle size distribution of the obtained microencapsulated chokeberry and bilberry powders was determined and quantified by a Mastersizer 2000 analyzer (Malvern Instruments, Worcestershire, UK). The parameters d_10_, d_50_, and d_90_, which represent the sizes of 10%, 50%, and 90% particles smaller than the remaining particles, were examined. The indicator of the size distribution width was defined as SPAN values (or PSD) calculated as (d_90_ − d_10_)/d_50_. D [3.2] represented the surface weighted mean, and D [4.3] represented the volume weighted mean, and the uniformity of the microparticles was verified as well.

#### 3.6.2. FTIR Spectroscopy Analysis

Fourier-transform infrared (FTIR) spectral analysis of the obtained powders (dried extracts, biopolymers, and microencapsulates) was observed in the range mode of 400 and 4000 cm^−1^ with a resolution of 4 cm^−1^ using a Nicolet iS10 (Thermo Scientific, Stockholm, Sweden) spectrometer. The FTIR spectral range was measured directly on the applied samples.

#### 3.6.3. Differential Scanning Calorimetry (DSC)

Thermal characteristics of chokeberry and bilberry microencapsulates were analyzed by the DSC131 Evo (SETARAM Instrumentation, Caluire-et-Cuire, France). The samples were positioned in aluminum pans (30 µL), followed by hermetical sealing. An empty pan was used as a blind probe. The heating profile was set as follows: both pans (reference and samples) were stabilized at 20 °C for 5 min and then heated to 350 °C with a heating rate of 10 °C/min and a nitrogen flow of 20 mL/min. A baseline run was accomplished using empty pans under the same conditions, whereas baseline subtraction and enthalpy determination (J/g) were carried out by CALISTO PROCESSING software version 1.38 equipped with SETARAM instrumentation.

### 3.7. Chemical Analysis of Microencapsulates

#### 3.7.1. Analysis of Total Phenolics

The content of total phenolic compounds in the bilberry and chokeberry leaves microencapsulates was assessed employing a previously established spectrophotometric method with Folin–Ciocalteu (FC) reagent with some modifications [114]. Diluted extract samples (200 µL) were mixed with 800 µL of sodium carbonate solution and 1000 µL of FC reagent and subsequently incubated for 2 h at room temperature. The quantification of total polyphenols was performed by measuring the absorbance at 765 nm. Experiments were conducted in triplicate, and the results are expressed as the mean value in milligrams of gallic acid equivalents (GAEs) per gram of dry weight of the drug (mg GAE/g DW).

#### 3.7.2. HPLC Analysis of Individual Compounds

Analyses were carried out on an Agilent 1260 RR HPLC instrument (Agilent, Waldbronn, Germany)-equipped diode-array detector working in the range of 190–550 nm. The samples were separated using a reverse-phase Zorbax SB-C18 (Agilent) analytical column (150 mm × 4.6 mm i.d.; 5 μm particle size). Mobile phase A was a 1% *v*/*v* solution of orthophosphoric acid in water; mobile phase B was acetonitrile. The gradient elution followed the following scheme: 0–2.6 min, 90–85% A; 2.6–8 min, 85% A; 8–10.8 min, 85–80% A; 10.8–18 min, 80% A; 18–23 min, 80–70% A; 23–25 min, 70–50% A; 25–27 min, 50–30% A; 27–29 min, 30–10% A; 29–31 min, 10–0% A; 31–34 min, 0%. The detection wavelengths were set at 260, 280, 320, and 360 nm, and the flow rate was 0.8 mL/min. The injection volume was 8 μL, and the column temperature was maintained at 40 °C. The identification of the compounds was achieved by comparing their UV spectra and retention time with those of authentic substances. The amounts of the compounds were calculated using calibration curves. The results are presented as micrograms per gram of dry weight (μg/g DW).

### 3.8. Biological Evaluation of Microcapsulates

#### 3.8.1. Radical Scavenging Activity—DPPH Assay

The radical scavenging activity of all prepared extracts was assessed using the 2,2-diphenyl-1-picrylhydrazyl (DPPH) assay [115]. The analysis was based on mixing 2 mL of test solution (5 different concentrations of the extract samples diluted in methanol) and 0.5 mL of 0.2 mg/mL freshly prepared methanol DPPH solution. The control sample was comprised of methanol (instead of the extract solution) and DPPH solution. The antioxidant activity of the test and control samples, including vitamin C as a reference standard, was measured spectrophotometrically at 517 nm after 30 min of incubation in the dark at room temperature. The obtained results were first expressed as the radical-scavenging capacity (RSC) via Equation (5) and further used to calculate the IC_50_ value.

RSC (%) = [(A_c_ − A_s_)/A_c_] × 100, (5)

A_s_ represents the absorbance of the samples at different concentrations, while A_c_ refers to the absorbance of the control.

#### 3.8.2. Evaluation of Hypoglycemic Activity

The α-amylase inhibitory activity of the dried extracts was tested according to the method reported by Ahmed et al. (2013) with minor modifications [116].

The sample solutions, the α-amylase enzyme solution (type VI-B, ≥5 units/mg solid), and the potato starch solution (1.0% (*w*/*v*)) were prepared in phosphate buffer (0.1 M, pH 6.9). The serial dilutions of the samples were mixed with the α-amylase solution and incubated at 37 °C for 15 min. Subsequently, the starch solution was added, and incubation was continued for 10 min. Finally, DNS (3,5-dinitrosalicylic acid) solution was added, and the samples were kept in a boiling water bath for 15 min. The control contained phosphate buffer instead of the plant extract. The absorbance was measured at 540 nm. The percentage inhibitions were calculated using Equation (6), where Ac is the absorbance of the control and As is the absorbance of the samples. The results are expressed as IC_50_ values. Acarbose was used as the reference standard.

Inhibition (%) = [(A_c_ − A_s_)/A_c_] × 100, (6)

The slightly modified method of Indrianingsih et al. (2015) was followed to testingα-glucosidase’s inhibitory activity for all dried extracts [117]. Extracts dissolved in phosphate buffer (0.1 M, pH 6.9) were treated with p-nitrophenyl-α-D-glucopyranoside solution and phosphate buffer. The mixtures were incubated at 37 °C for 5 min. Then, α-glucosidase enzyme solution (≥10 units/mg solid) was added, and the incubation was continued for 15 min. The reaction was stopped by adding 0.2 M sodium carbonate solution. The control contained phosphate buffer instead of plant extract. The absorbance was measured at 400 nm. The percentage inhibitions were calculated using Equation (6).

#### 3.8.3. Antimicrobial Assay

The antimicrobial activity of all dried extracts was tested against the most common microorganisms that cause foodborne or skin infections. Among the pathogens commonly recognized as causative agents of foodborne infections, the following bacteria were used: *Escherichia coli* O157:H7, *Salmonella enterica* serovar Typhimurium ATCC 14028, *Shigella flexneri* ATCC 12022, *Listeria monocytogenes* ATCC 19114, and *Enterococcus faecalis* ATCC 29212. To examine the antimicrobial activity against the most common skin infection pathogens, several bacteria and fungi also applied in the cosmetology “Challenge test” were used: the bacteria *Escherichia coli* ATCC 8739, *Pseudomonas aeruginosa* ATCC 27853, *Staphylococcus epidermidis* ATCC 12228, and *Staphylococcus aureus* ATCC 25923; the yeast *Candida albicans* ATCC 10231; and the mold *Aspergillus brasiliensis* ATCC 16404.

Antimicrobial activity was evaluated with the resazurin microdilution method according to the recommendations of the National Committee for Clinical Laboratory Standards (CLSI, 2002) [118]. All bacterial strains were sub-cultured on Mueller–Hinton Agar (MHA), while *C. albicans* and *A. brasiliensis* were grown on Sabouraud dextrose agar (SAB) and potato dextrose agar, respectively. For further preparation, bacterial strains were cultivated overnight in Mueller–Hinton Broth (MHB), and for fungi, slopes were flooded with 0.85% saline, and the conidia were gently probed. The remaining dilutions in sterile saline were obtained to achieve final concentrations of 1 × 10^6^ CFU/mL for bacteria and 2 × 10^4^ CFU/mL for fungi. Briefly, serial dilutions of dry extracts in MHB (for bacteria) or Tryptone Soya Broth (TSB) were prepared on a microtiter plate with 96 wells. Additionally, 10 μL of microbial suspension and 10 μL of resazurin solution, an indicator of growth, were added to reach a final concentration of 100 μL per well. Microbial strains in an appropriate liquid medium with resazurin solution were used as positive controls, while negative controls contained only the medium with the indicator. After the incubation of plates containing bacteria for 24 h at 37 °C and the incubation of fungi-containing trays for three to seven days at 25 °C, the minimum inhibitory concentrations (MICs) were determined as the lowest concentration without the visible growth, detected as no change in the well color. Cell growth was detected in wells with pink color due to the reduction of purple resazurin to pink resorufin by the activity of living microbial cells. An appropriate volume of wells without indicated growth was inoculated in sterile liquid media or SAB agar, depending on the type of microorganism. The minimum bactericidal concentration (MBC) was determined after 24 h of incubation at 37 °C, and the minimum fungicidal concentration (MFC) was determined after 25 °C incubation for 3 days, as the lowest concentration without visible growth. All tests were performed in triplicate.

#### 3.8.4. Photoprotective Activity

The photoprotective potentials of bilberry and chokeberry leaf dry extracts were determined in vitro according to the method described by Oliveira et al. (2021) [119]. To express the photoprotective activity, the sun protection factor (SPF) was calculated. The extract solutions were prepared in 50% ethanol (*v*/*v*) at concentrations of 0.025, 0.05, and 0.1 mg/mL. The absorption of each solution was measured in triplicate at 290–320 nm, with 5 nm increments. The blank was 50% ethanol (*v*/*v*). The SPF was calculated using Equation (7), where CF is the correction factor (equal to 10); EE (λ) is the erythemal effect spectrum; I (λ) is the solar intensity spectrum, and Abs (λ) is the absorbance of the solution at a wavelength (λ). The values of EE × I are constants and were determined by Sayre et al. (1979) [120].

SPF = CF × 320 ∑290 EE (λ)I(λ)Abs(λ),(7)

### 3.9. Statistical Analysis

One-way analysis of variance (ANOVA) was applied to elucidate significant differences between means, determined at *p* < 0.05. The significance of differences between means was assessed using Duncan’s MRL post hoc tests. Values followed by different letters in the same column were considered significantly different. Pearson’s correlation coefficient (*r*) was used to reveal the degree of linear dependence between the bioactive components of bilberry and chokeberry leaf microencapsulates and the observed biological activity. The correlation was considered statistically significant if the *p*-value was below 0.05. All the statistical analyses were performed using Statistica software package version 5.0 (StatSoft Co., Tulsa, OK, USA).

## 4. Conclusions

The current investigation aimed to develop a feasible method for producing microencapsulated powders with chokeberry and bilberry leaves using pectin and HP-β-CD as encapsulating carriers. The obtained micro-sized powders were assessed regarding technological and physicochemical characteristics, demonstrating a powder yield from 61.99 to 67.86% for chokeberry powders and from 67.87 to 73.27% for bilberry powders. The addition of pectin increased the powder yield, while the addition of HP-β-CD slightly influenced the yield of the bilberry powder, both with satisfactory moisture content. The addition of biopolymers in extracts improved the flowability and cohesive properties, while HP-β-CD significantly shortened the rehydration time. The microencapsulated powders obtained with pectin and pectin/HP-β-CD as wall materials exhibited slightly higher particle sizes in comparison with the carrier-free samples. We performed an FTIR analysis, which suggested that the microencapsulation process occurred on the surface domain through physical interactions, while a DSC analysis indicated the great potential of the carriers used to protect the spray-dried particles against temperature degradation. The quickly rising prevalence of diabetes worldwide increases the need for novel and alternative therapies for managing and preventing diabetes-related complications more efficiently and with fewer side effects. Chokeberry and bilberry leaves have demonstrated great potential as valuable sources of potent natural antioxidants with additional hypoglycemic, antibacterial, and anti-inflammatory effects. The present study showed that the chokeberry and bilberry leaf extract powders are a rich source of polyphenolic compounds, with chlorogenic acid and isoquercetin being the most abundant. The examined microparticles showed strong scavenging activity in the DPPH assay and a great potential to suppress hyperglycemia through the inhibition of the α-amylase and α-glucosidase enzymes. Microbiological evaluation additionally revealed that they exhibited significant bactericidal activity at lower concentrations against Gram-positive bacteria, especially *S. aureus* and *S. epidermidis*. Furthermore, the examined powders were characterized as promising naturally occurring photoprotective agents. Overall, the spray-drying microencapsulation of chokeberry and bilberry leaf extracts with pectin and pectin/HP-β-CD as wall materials opens a new perspective for the preservation and stabilization of valuable antioxidants with potent biological activities, which can contribute to consumers’ well-being by being incorporated into new pharmaceuticals or functional food products.

## Figures and Tables

**Table 1:** Technological parameters of bilberry and chokeberry leaf extracts and their microencapsulates.

Samples	Yield (%)	Moisture Content (%)	Bulk Density (g/mL)	Tapped Density (g/mL)	CI	HR	pH	Rehydration (s)
B	61.99	3.35 ± 0.21 ^a^	0.21 ± 0.00 ^b^	0.31 ± 0.01 ^b^	29.68 ± 1.11 ^b^	1.42 ± 0.02 ^b^	4.12 ± 0.01 ^a^	72.72 ± 1.52 ^b^
BP	66.20	3.91 ± 0.36 ^a^	0.29 ± 0.01 ^a^	0.36 ± 0.00 ^a^	19.09 ± 0.91 ^c^	1.24 ± 0.02 ^c^	4.00 ± 0.01 ^b^	95.52 ± 1.79 ^a^
BPCD	67.86	3.54 ± 0.15 ^a^	0.28 ± 0.02 ^a^	0.41 ± 0.04 ^a^	32.58 ± 0.75 ^a^	1.49 ± 0.02 ^a^	3.88 ± 0.03 ^c^	29.27 ± 0.32 ^c^
C	72.11	3.12 ± 0.03 ^b^	0.20 ± 0.01 ^c^	0.34 ± 0.03 ^a^	40.07 ± 1.61 ^a^	1.67 ± 0.04 ^a^	4.50 ± 0.02 ^a^	21.48 ± 0.89 ^c^
CP	73.27	3.41 ± 0.06 ^a^	0.27 ± 0.01 ^a^	0.33 ± 0.02 ^a^	17.09 ± 1.09 ^b^	1.21 ± 0.02 ^b^	4.42 ± 0.03 ^b^	80.22 ± 1.93 ^a^
CPCD	67.87	2.95 ± 0.02 ^c^	0.22 ± 0.02 ^b^	0.38 ± 0.04 ^a^	38.59 ± 2.23 ^a^	1.63 ± 0.06 ^a^	4.49 ± 0.04 ^ab^	70.12 ± 0.63 ^b^

Different letters within a column indicate a significant difference between samples at *p* < 0.05. B—bilberry extract; BP—bilberry extract with pectin; BPCD—bilberry extract with pectin and HP-β-CD; C—chokeberry extract; CP—chokeberry extract with pectin; CPCD—chokeberry extract with pectin and HP-β-CD.

**Table 2:** The particle size of bilberry and chokeberry leaf extracts and their microencapsulates.

Samples	d_10_ *	d_50_ **	d_90_	Span/PDI ***	D (4.3)	D (3.2)	Uniformity
B	1.34 ± 0.08 ^b^	4.74 ± 0.59 ^abc^	9.58 ± 0.64 ^bc^	1.74 ± 0.20 ^b^	5.87 ± 0.32 ^cd^	2.46 ± 0.19 ^ab^	0.66 ± 0.06 ^cd^
BP	1.78 ± 0.13 ^a^	5.94 ± 0.75 ^a^	31.97 ± 5.14 ^a^	5.09 ± 0.50 ^a^	13.76 ± 1.53 ^a^	3.06 ± 0.25 ^a^	1.76 ± 0.19 ^b^
BPCD	1.79 ± 0.28 ^a^	5.79 ± 0.48 ^ab^	15.24 ± 1.89 ^b^	2.32 ± 0.19 ^b^	9.90 ± 1.27 ^b^	3.08 ± 0.30 ^a^	2.32 ± 0.17 ^a^
C	0.99 ± 0.13 ^b^	3.83 ± 0.21 ^c^	7.41 ± 0.68 ^c^	1.68 ± 0.26 ^b^	4.11 ± 0.30 ^d^	2.06 ± 0.26 ^b^	0.49 ± 0.07 ^d^
CP	1.12 ± 0.09 ^b^	4.23 ± 0.26 ^c^	8.87 ± 0.45 ^c^	1.83 ± 0.11 ^b^	6.34 ± 0.59 ^cd^	2.24 ± 0.19 ^b^	0.93 ± 0.10 ^c^
CPCD	1.11 ± 0.07 ^b^	4.49 ± 0.51 ^bc^	9.54 ± 0.70 ^bc^	1.88 ± 0.30 ^b^	6.88 ± 0.52 ^c^	2.27 ± 0.38 ^b^	0.96 ± 0.06 ^c^

* d_10_, d_50_, and d_90_ represent the sizes where 10%, 50%, and 90% of the particles are smaller than the remaining particles in µm; ** mean diameter size; *** calculated as (d_90_ − d_10_)/d_50_; B—bilberry extract; BP—bilberry extract with pectin; BPCD—bilberry extract with pectin and HP-β-CD; C—chokeberry extract; CP—chokeberry extract with pectin; CPCD—chokeberry extract with pectin and HP-β-CD. Means followed by the same letter within the same column are not significantly different according to Duncan’s test (α = 0.05%).

**Table 3:** The transition temperatures and enthalpy changes of bilberry and chokeberry leaf extracts and their microencapsulates.

Samples	T1 (°C)	T2 (°C)	∆H1 (J/g)	∆H2 (J/g)
C	105.02 ± 7.65 ^ab^	134.49 ± 12.58 ^c^	22.26 ± 2.86 ^e^	101.94 ± 12.24 ^b^
CP	109.23 ± 11.35 ^ab^	145.76 ± 7.47 ^bc^	47.36 ± 6.42 ^de^	122.97 ± 12.01 ^ab^
CPCD	114.52 ± 7.56 ^ab^	139.14 ± 19.12 ^c^	77.09 ± 8.83 ^cd^	135.38 ± 12.31 ^a^
B	84.58 ± 7.77 ^b^	137.33 ± 16.05 ^c^	44.96 ± 4.24 ^de^	59.87 ± 7.79 ^c^
BP	119.28 ± 15.76 ^a^	150.67 ± 19.29 ^bc^	98.14 ± 13.74 ^c^	105.28 ± 12.88 ^b^
BPCD	109.26 ± 14.12 ^ab^	170.11 ± 12.09 ^bc^	82.05 ± 8.84 ^c^	22.71 ± 1.58 ^d^
P	112.77 ± 5.66 ^ab^	187.59 ± 12.55 ^b^	245.59 ± 18.28 ^a^	45.18 ± 5.11 ^cd^
HP-β-CD	100.31 ± 14.20 ^ab^	328.27 ± 19.20 ^a^	177.93 ± 21.13 ^b^	48.91± 3.95 ^cd^

Means followed by different letters are significantly different according to Tukey’s post hoc test at *p* ≤ 0.05. B—bilberry extract; BP—bilberry extract with pectin; BPCD—bilberry extract with pectin and HP-β-CD; C—chokeberry extract; CP—chokeberry extract with pectin; CPCD—chokeberry extract with pectin and HP-β-CD; P—pectin.

**Table 4:** Content of total phenolic compounds and antioxidant activity in bilberry and chokeberry leaf extracts and their microencapsulates.

Sample	TPC (mg GAE/g DW)	DPPH IC_50_ (µg/mL)	Sample	TPC (mg GAE/g DW)	DPPH IC_50_ (µg/mL)
B	367.76 ± 28.14 ^a^	9.14 ± 0.20 ^b^	C	227.59 ± 0.92 ^a^	20.36 ± 0.36 ^c^
BP	333.72 ± 4.51 ^ab^	11.02 ± 0.06 ^a^	CP	186.85 ± 8.59 ^b^	22.04 ± 0.17 ^b^
BPCD	323.35 ± 16.77 ^b^	10.79 ± 0.04 ^a^	CPCD	192.92 ± 11.55 ^b^	27.01 ± 0.52 ^a^

Different letters within a column indicate a significant difference between samples at *p* < 0.05. B—bilberry extract; BP—bilberry extract with pectin; BPCD—bilberry extract with pectin and HP-β-CD; C—chokeberry extract; CP—chokeberry extract with pectin; CPCD—chokeberry extract with pectin and HP-β-CD; TPC—total phenolic content.

**Table 5:** The content of individual polyphenolics of bilberry and chokeberry leaf extracts and their microencapsulates.

Sample	Chlorogenic Acid	Quercitrin	Quercetin	Hyperoside	Rutin	Isoquercetin
µg/g DW						
B	54.01 ± 4.70 ^a^	7.48 ± 0.66 ^a^	0.19 ± 0.02 ^a^	11.12 ± 0.56 ^a^	10.11 ± 0.89 ^a^	40.25 ± 3.53 ^a^
BP	50.71 ± 4.31 ^a^	7.04 ± 0.57 ^a^	0.09 ± 0.01 ^b^	10.17 ± 1.00 ^ab^	9.67 ± 0.79 ^a^	38.47 ± 2.33 ^a^
BPCD	48.27 ± 4.63 ^a^	6.52 ± 0.62 ^a^	0.04 ± 0.00 ^c^	9.54 ± 0.64 ^b^	9.38 ± 0.51 ^a^	37.20 ± 2.94 ^a^
C	40.05 ± 3.24 ^a^	6.50 ± 0.51 ^a^	0.31 ± 0.02 ^a^	6.35 ± 0.49 ^a^	8.64 ± 0.77 ^a^	11.37 ± 0.79 ^a^
CP	36.40 ± 2.00 ^ab^	5.78 ± 0.38 ^ab^	0.28 ± 0.03 ^a^	5.66 ± 0.52 ^ab^	8.04 ± 0.43 ^a^	10.81 ± 0.95 ^a^
CPCD	34.67 ± 1.78 ^b^	5.35 ± 0.48 ^b^	0.22 ± 0.01 ^b^	5.32 ± 0.47 ^b^	7.77 ± 0.70 ^a^	10.70 ± 0.67 ^a^

Different letters within a column indicate a significant difference between samples at *p* < 0.05. B—bilberry extract; BP—bilberry extract with pectin; BPCD—bilberry extract with pectin and HP-β-CD; C—chokeberry extract; CP—chokeberry extract with pectin; CPCD—chokeberry extract with pectin and HP-β-CD.

**Table 6:** Pearson’s correlation coefficients between the content of total and individual phenolics and DPPH antioxidant activity of bilberry and chokeberry leaf extracts and their microencapsulates.

	Chlorogenic Acid	Rutin	Hyperoside	Isoquercetin	Quercetin	Quercitrin	TPC
**DPPH**	−0.9771 *	−0.9728 *	−0.9734 *	−0.9574 *	−0.6917	−0.9080 *	−0.9655 *

Values marked with “*” are significant at *p* < 0.05.

**Table 7:** Hypoglycemic activity of bilberry and chokeberry leaf extracts and their microencapsulates.

Sample	α-amylase IC_50_ (mg/mL)	α-glucosidase IC_50_ (µg/mL)
B	4.30 ± 0.22 ^a^	12.50 ± 1.13 ^b^
BP	4.50 ± 0.27 ^a^	18.36 ± 1.72 ^a^
BPCD	4.70 ± 0.41 ^a^	19.59 ± 1.92 ^a^
C	8.66 ± 0.64 ^a^	5.00 ± 0.31 ^b^
CP	9.59 ± 0.84 ^a^	10.79 ± 1.06 ^a^
CPCD	10.04 ± 0.95 ^a^	12.29 ± 1.16 ^a^
Acarbose	2.06 ± 0.13	156.64 ± 16.63

Different letters within a column indicate a significant difference between samples at *p* < 0.05. B—bilberry extract; BP—bilberry extract with pectin; BPCD—bilberry extract with pectin and HP-β-CD; C—chokeberry extract; CP—chokeberry extract with pectin; CPCD—chokeberry extract with pectin and HP-β-CD.

**Table 8:** Minimum inhibitory concentration (MIC) of bilberry and chokeberry leaf extracts and their microencapsulates on foodborne bacteria.

Sample	Gram-Positive		Gram-Negative		
	*E. faecalis*	*L. monocytogenes*	*E. coli*	*S*. Typhimurium	*S. flexneri*
B	2.5	30	30	40	5
BP	2.5	40	40	50	7.5
BPCD	2.5	40	40	50	7.5
C	10	30	30	40	40
CP	15	50	50	60	40
CPCD	15	40	50	80	40

MIC values are expressed as mg/mL. B—bilberry extract; BP—bilberry extract with pectin; BPCD—bilberry extract with pectin and HP-β-CD; C—chokeberry extract; CP—chokeberry extract with pectin; CPCD—chokeberry extract with pectin and HP-β-CD.

**Table 9:** Minimum inhibitory concentration (MIC) of bilberry and chokeberry leaf extracts and their microencapsulates on microorganisms that cause skin infections and cosmetic product spoilage.

Sample	Gram-Positive		Gram-Negative		Fungi	
	*S. aureus*	*S. epidermidis*	*E. coli*	*P. aeruginosa*	*C. albicans*	*A. brasiliensis*
B	1.75	1.75	5	40	20	10
BP	2.5	2.5	7.5	70	40	20
BPCD	2.5	2.5	5	70	30	20
C	5	5	10	40	20	10
CP	7.5	7.5	10	80	40	20
CPCD	5	5	10	60	40	20

MIC values are expressed as mg/mL. B—bilberry extract; BP—bilberry extract with pectin; BPCD—bilberry extract with pectin and HP-β-CD; C—chokeberry extract; CP—chokeberry extract with pectin; CPCD—chokeberry extract with pectin and HP-β-CD.

**Table 10:** Correlation between antimicrobial activity and polyphenols of bilberry and chokeberry leaf extracts and their microencapsulates.

Antimicrobial Activity	Total Phenols	Chlorogenic Acid	Rutin	Hyperoside	Isoquercetin	Quercetin	Quercitrin
*E. coli* ^1^	0.5257	0.5571	0.6252	0.4861	0.3437	0.1280	0.7580
*S*. Typhimurium	0.5938	0.6449	0.7112	0.5738	0.4417	0.0272	0.8372 *
*S. flexneri*	0.9735 *	0.9652 *	0.9452 *	0.9782 *	0.9633 *	−0.6673	0.8561 *
*E. faecalis*	0.9763 *	0.9617 *	0.9382 *	0.9776 *	0.9965 *	−0.8343 *	0.8216 *
*L. monocytogenes*	0.4072	0.4129	0.4745	0.3505	0.2150	0.2021	0.5964
*E. coli* ^2^	0.8664 *	0.8344 *	0.8126 *	0.8544 *	0.8694 *	−0.6884	0.6802
*P. aeruginosa*	0.2318	0.2474	0.3135	0.1818	0.0307	0.4151	0.4669
*S. aureus*	0.9689 *	0.9528 *	0.9380 *	0.9613 *	0.9363 *	−0.6258	0.8585 *
*S. epidermidis*	0.9689 *	0.9528 *	0.9380 *	0.9613 *	0.9363 *	−0.6258	0.8585 *
*C. albicans*	0.3535	0.3770	0.4467	0.3082	0.1652	0.2882	0.5808
*A. brasiliensis*	0.2519	0.2899	0.3634	0.2160	0.0516	0.4511	0.5392

^1^, foodborne; ^2^, skin; values marked with “*” are significant at *p* < 0.05.

**Table 11:** Photoprotective activity of bilberry and chokeberry leaf extracts and their microencapsulates.

Sample	Sun Protection Factor (SPF)		
Concentration (mg/mL)	0.025	0.05	0.1
B	2.60 ± 0.02 ^a^	5.12 ± 0.12 ^a^	9.74 ± 0.28 ^a^
BP	2.38 ± 0.09 ^b^	4.66 ± 0.18 ^b^	9.10 ± 0.36 ^ab^
BPCD	2.16 ± 0.06 ^c^	4.23 ± 0.12 ^c^	8.22 ± 0.16 ^b^
C	2.04 ± 0.11 ^a^	4.04 ± 0.23 ^a^	7.90 ± 0.38 ^a^
CP	2.09 ± 0.09 ^a^	4.05 ± 0.08 ^a^	7.81 ± 0.25 ^a^
CPCD	1.91 ± 0.03 ^b^	3.66 ± 0.16 ^b^	6.97 ± 0.26 ^a^

Different letters within a column indicate a significant difference between samples at *p* < 0.05. B—bilberry extract; BP—bilberry extract with pectin; BPCD—bilberry extract with pectin and HP-β-CD; C—chokeberry extract; CP—chokeberry extract with pectin; CPCD—chokeberry extract with pectin and HP-β-CD.

## Data Availability

The data is contained within the manuscript and Supplementary Materials.

## Supplementary Materials

The following supporting information can be downloaded at: https://www.mdpi.com/article/10.3390/plants12233979/s1, Table S1: Minimum bactericidal concentration (MBC) and minimum fungicidal concentration (MFC) of bilberry and chokeberry leaf extracts and their microencapsulates on foodborne and skin bacteria.
