# Supplementary material for: Microencapsulated Bilberry and Chokeberry Leaf Extracts with Potential Health Benefits
Source: Plants (Basel). 2023 Nov 27;12(23):3979. doi: 10.3390/plants12233979 (PMC10707773; doi:10.3390/plants12233979)
Supplement: Supplementary file 1 [file plants-12-03979-s001.zip › plants-2706982-supplementary.pdf]

Supplementary material

Table S1. Minimum bactericidal concentration (MBC) and minimum fungicidal concentration (MFC) of bilberry and chokeberry leaves extracts and their microencapsulates on foodborne and skin bacteria.

		**Samples** MBC/MFC (mg/mL)					
	**Microorganism**	B	BP	BPCD	C	CP	CPCD
foodborne	*E. faecalis*	5	5	5	15	15	15
	L. monocytogenes	40	80	60	>80	80	80
	E. coli	40	80	60	>80	>80	80
	S. Typhimurium	80	>80	>80	>80	>80	>80
	S. flexneri	7.5	10	10	40	60	50
skin	*S. aureus*	2.5	5	5	7.5	12.5	7.5
	S. epidermidis	5	5	5	7.5	12.5	10
	E. coli	7.5	7.5	7.5	10	12.5	12.5
	P. aeruginosa	65	70	70	>80	>80	>80
	C. albicans	35	40	40	40	50	50
	A. brasiliensis	30	40	43	35	40	30

MBC and MFC values are expressed as mg/mL. B - bilberry extract; BP, bilberry extract with pectin; BPCD - bilberry extract with pectin and hydroxypropyl-β-cyclodextrin (HP-β-CD); C - chokeberry extract; CP - chokeberry extract with pectin; CPCD - chokeberry extract with pectin and HP-β-CD.
